# Supplementary material for: Phosphoproteomics of osimertinib-tolerant persister cells reveals targetable kinase-substrate signatures
Source: Mol Syst Biol. 2025 Sep 29;21(11):1547–62. doi: 10.1038/s44320-025-00141-1 (PMC12583488; doi:10.1038/s44320-025-00141-1)
Supplement: Supplementary file 1 — Appendix [file 44320_2025_141_MOESM1_ESM.pdf]

Appendix for

# Phosphoproteomics of Osimertinib-Tolerant Persister Cells Reveals Targetable Kinase-Substrate Signatures

Hsiang-En Hsu[1,5], Matthew J. Martin[2,5], Shao-Hsing Weng[1], Reta Birhanu Kitata[1], Srikar Nagelli[2], Chiung-Yun Chang[3], Sonja Hess[3]*, Yu-Ju Chen[1,4]*

[1] Institute of Chemistry, Academia Sinica, Taipei, Taiwan
[2] Research and Early Development, Oncology R&D, AstraZeneca, Cambridge, UK
[3] Dynamic Omics, Centre for Genomics Research, Discovery Sciences, BioPharmaceuticals R&D, AstraZeneca, Gaithersburg, MD, USA
[4] Department of Chemistry, National Taiwan University, Taipei, Taiwan
[5] These authors contributed equally
* Correspondence: Sonja.Hess@astrazeneca.com (S.H.), yujuchen@as.edu.tw (Y.-J.C.)

## Table of Contents

Appendix Figure S1: Page 2
Appendix Figure S2: Page 3
Appendix Figure S3: Page 4
Appendix Figure S4: Page 5
Appendix Figure S5: Page 6
Appendix Figure S6: Page 7
Appendix Figure S7: Page 8
Appendix Figure S8: Page 9
Appendix Figure S9: Page 10
Appendix Figure S10: Page 11

# Appendix Figure S1

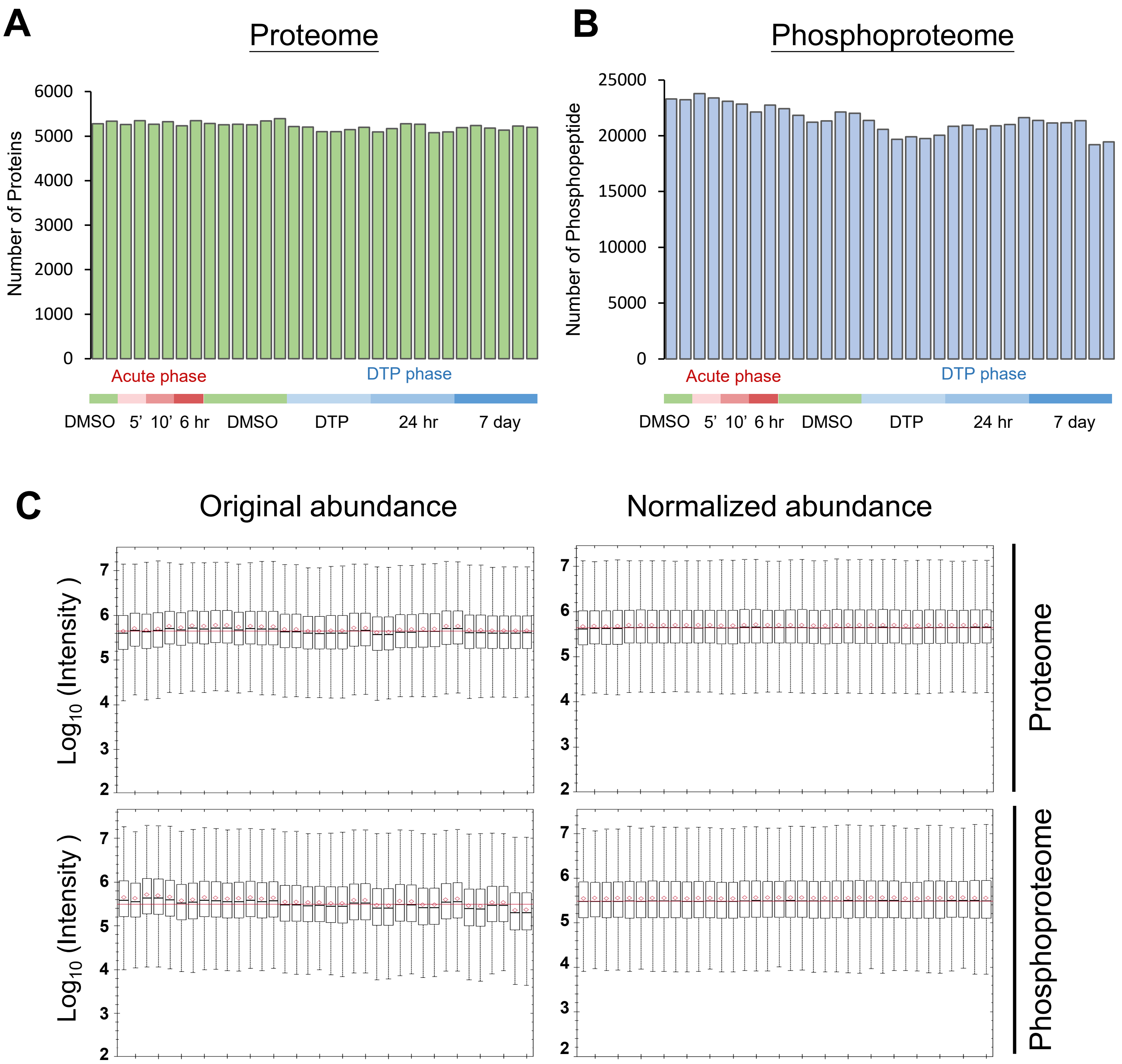


**Appendix Figure S1. Proteome and phosphoproteome quantification across different time-point sample batches.** (A) Protein identifications across different indicated time-points. (B) Phosphopeptide identifications across different indicated time-points. (C) The abundance of proteins and phosphopeptides were normalized by Spectronaut.

# Appendix Figure S2

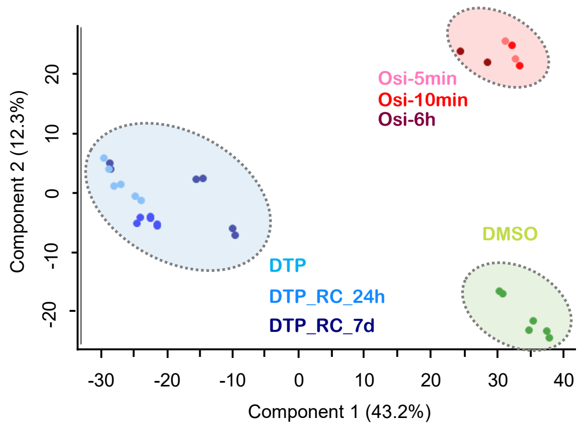


**Appendix Figure S2. PCA plot of different time-point of phosphoproteome.**
The control samples (DMSO), acute phase, and DTP phase are well separated.

# Appendix Figure S3

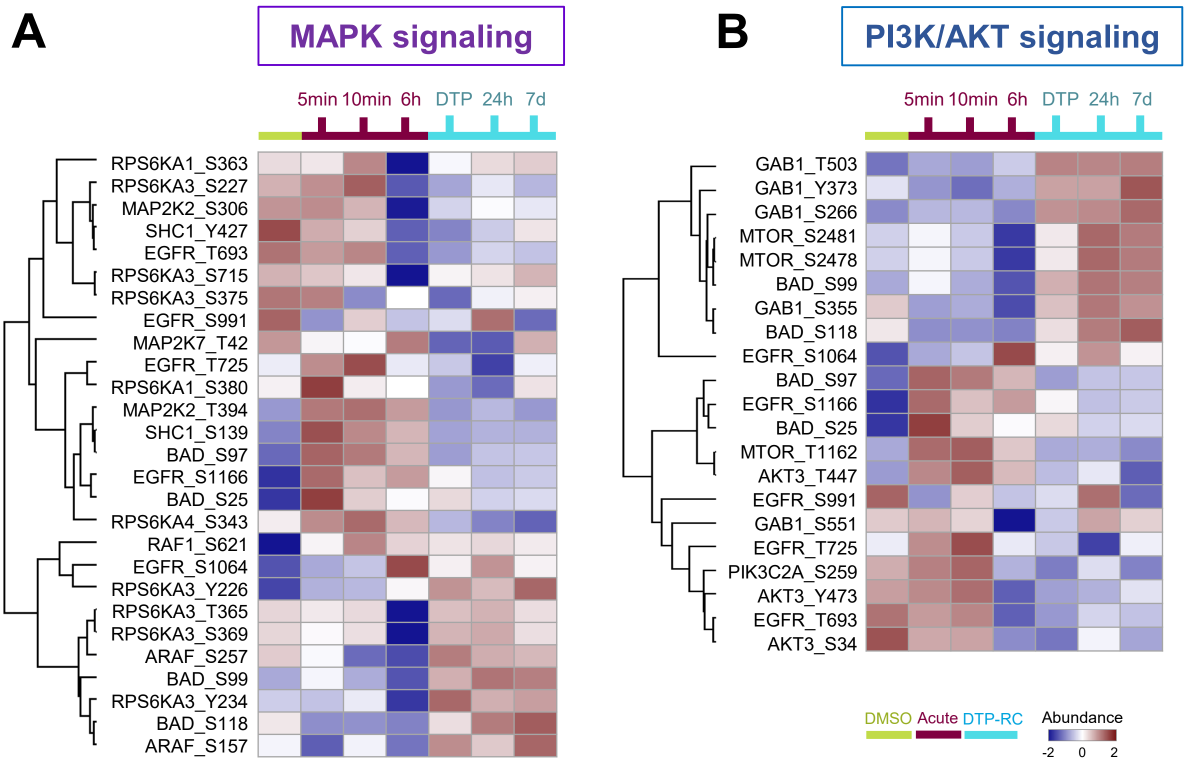


**Appendix Figure S3. The phosphorylation site expression levels.** (A) Heatmap showing the expression levels of phosphorylation sites involved in the MAPK signaling pathway. (B) Heatmap showing the expression levels of phosphorylation sites involved in the PI3K/AKT signaling pathway.

# Appendix Figure S4

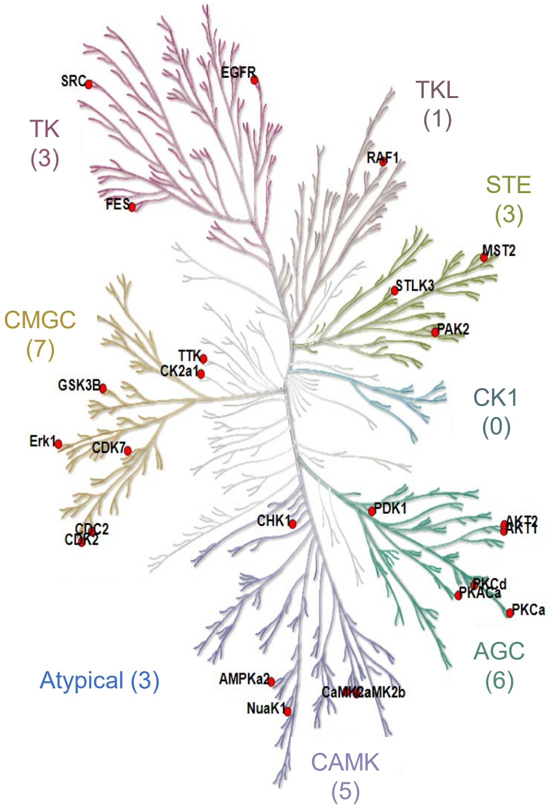


**Appendix Figure S4. Kinase tree analysis.** Kinase tree for mapping 28 kinases from the top 50 phosphoproteome expression profiling in DTP phase. The kinase families listed includes TK (tyrosine kinases), TKL (tyrosine kinase-like), CK1 (casein kinase 1), CAMK (calcium/calmodulin- dependent protein kinase), AGC (containing PKA, PKG, PKC families), CMGC (containing CDKs, MAPK, GSK, CLK families), and STE (serine/threonine kinases many involved in MAPK kinases cascade).

# Appendix Figure S5

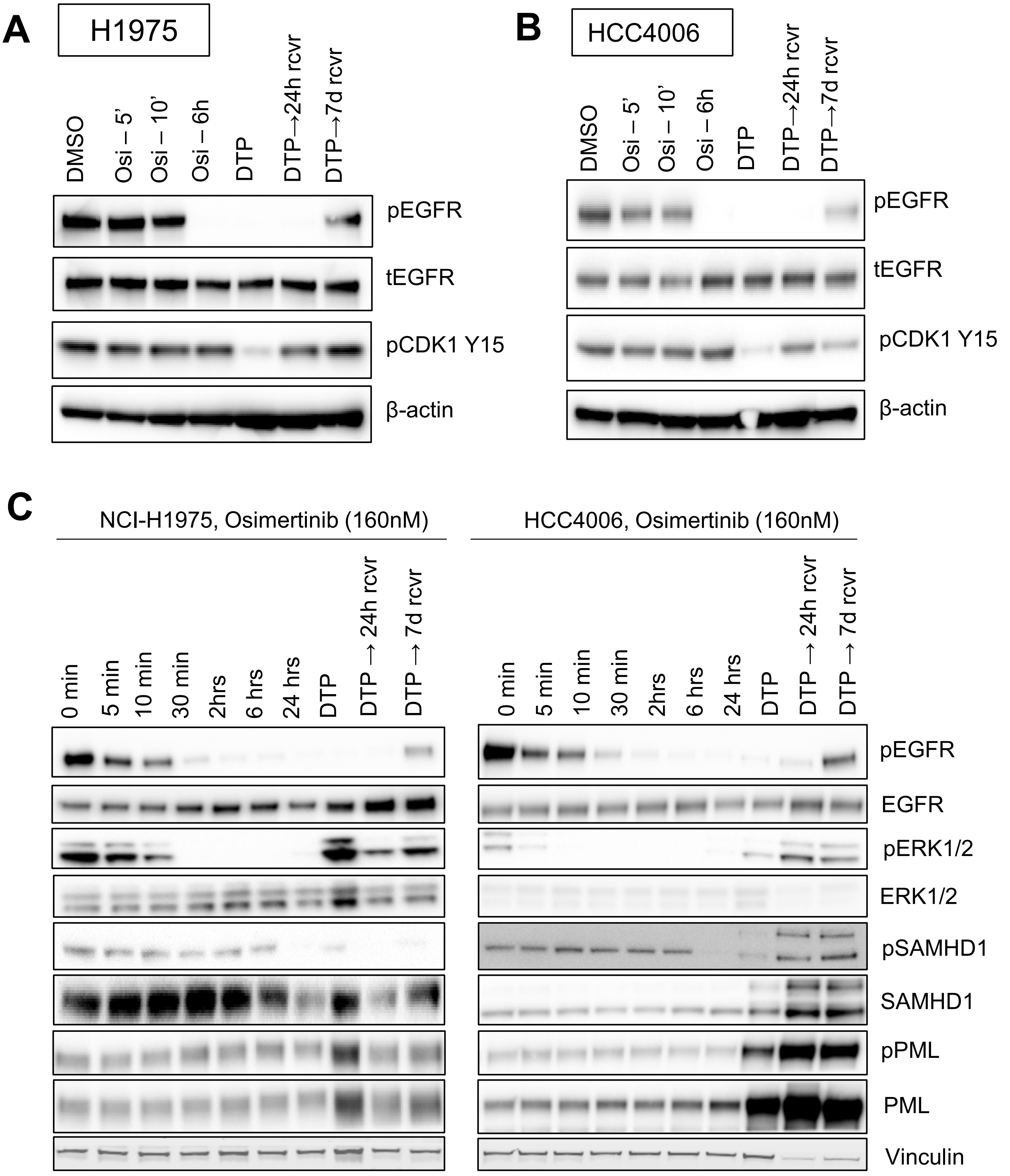


**Appendix Figure S5. Analysis of CDK1 pathway signaling endpoints.** (A to C) NCI-H1975 and HCC4006 cells were treated with Osimertinib 160 nM for the indicated time points, lysed and subjected to western blotting using the indicated antibodies. DTP-> 24h rcvr and DTP -> 7d rcvr indicate 24h and 7d of recovery from DTP state, respectively, where fresh media without osimertinib was added to the cells.

# Appendix Figure S6

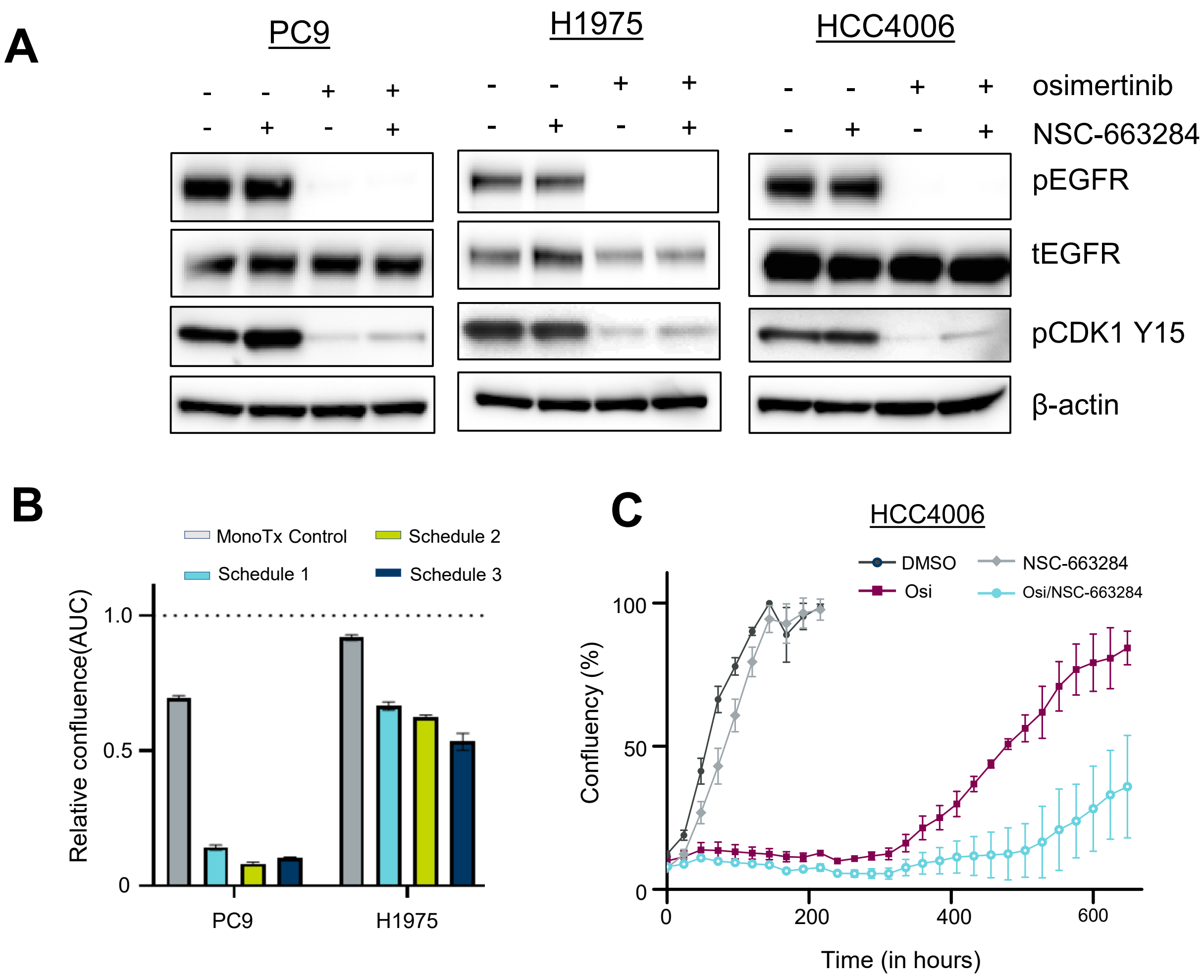


**Appendix Figure S6. Effect of CDK1 inhibition on DTP signaling and growth.**
(A) Western blotting against the indicated proteins in the PC9, H1975 and HCC4006 cell line treated with Osimertinib (160 nM) and/or NSC-663284 (1 μM) for 24h. (B) PC9 and H1975 were treated long-term with the varying schedules of osimertinib (160 nM) and/or NSC-663284 (1 μM) , as described in figure 6C. Growth was assessed as % confluence as measured by the Incucyte imaging platform. Bar graphs represent normalised AUC of the plotted confluence (n=3). (C) HCC4006 cells were treated continuously with Osimertinib (160 nM) NSC-663284 (1 μM) or the two drugs in combination, and growth was assessed as % confluence using the Incucyte imaging platform (n=3).

# Appendix Figure S7

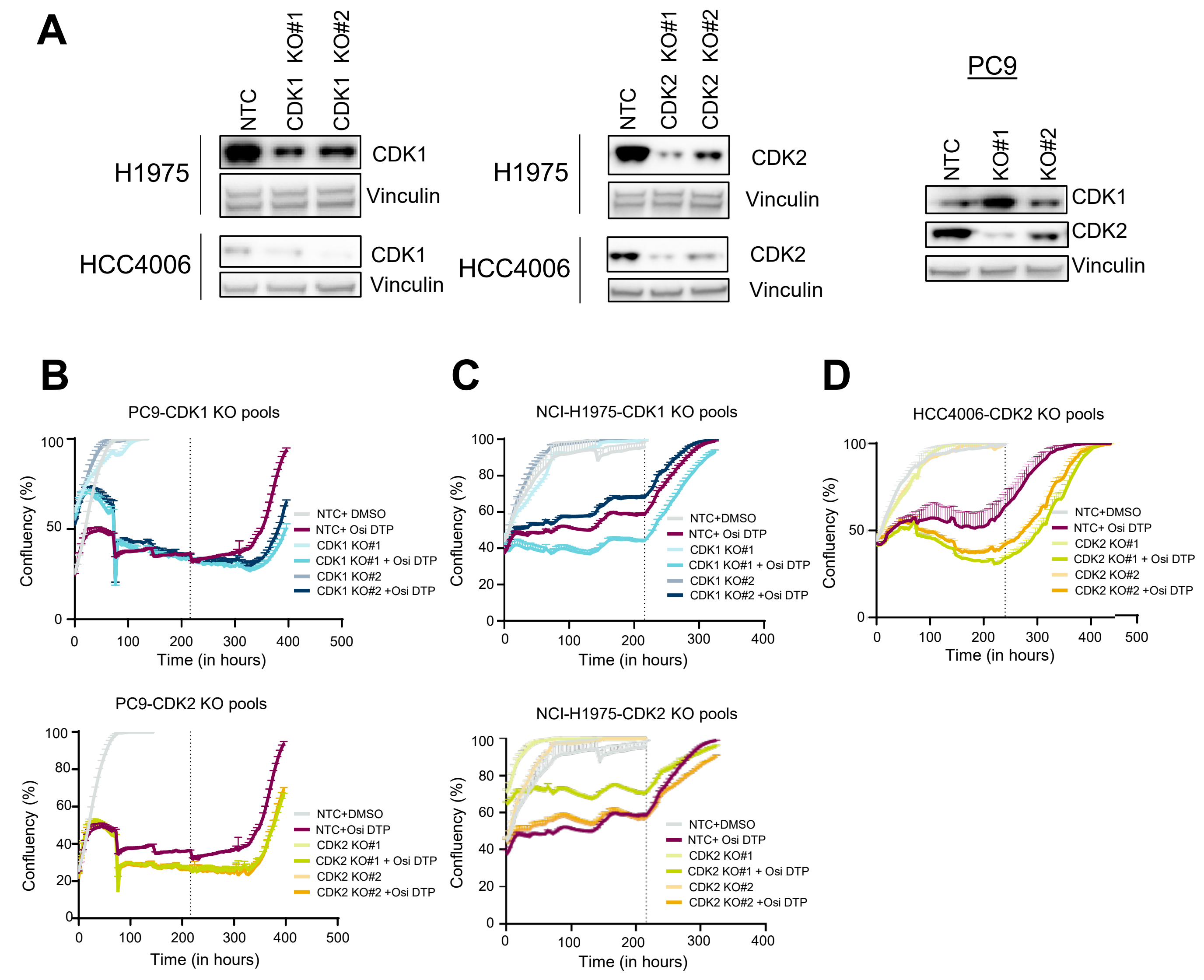


**Appendix Figure S7. Effect of CDK1/2 knockdown on DTP growth.** (A) Western blotting against the indicated proteins in the PC9, H1975 and HCC4006 cell lines transfected with CRISPR-targeting constructs to non-targeted control (NTC), CDK1 or CDK2. (B)-(D) Cell confluence plots for PC9, H1975 and HCC4006 cells transfected with CRISPR constructs targeting NTC, CDK1 or CDK2 and treated with 160 nM Osimertinib for 10 days, followed by drug washout. Growth was assessed as % confluency measured by the incucyte imaging platform (n=3).

# Appendix Figure S8

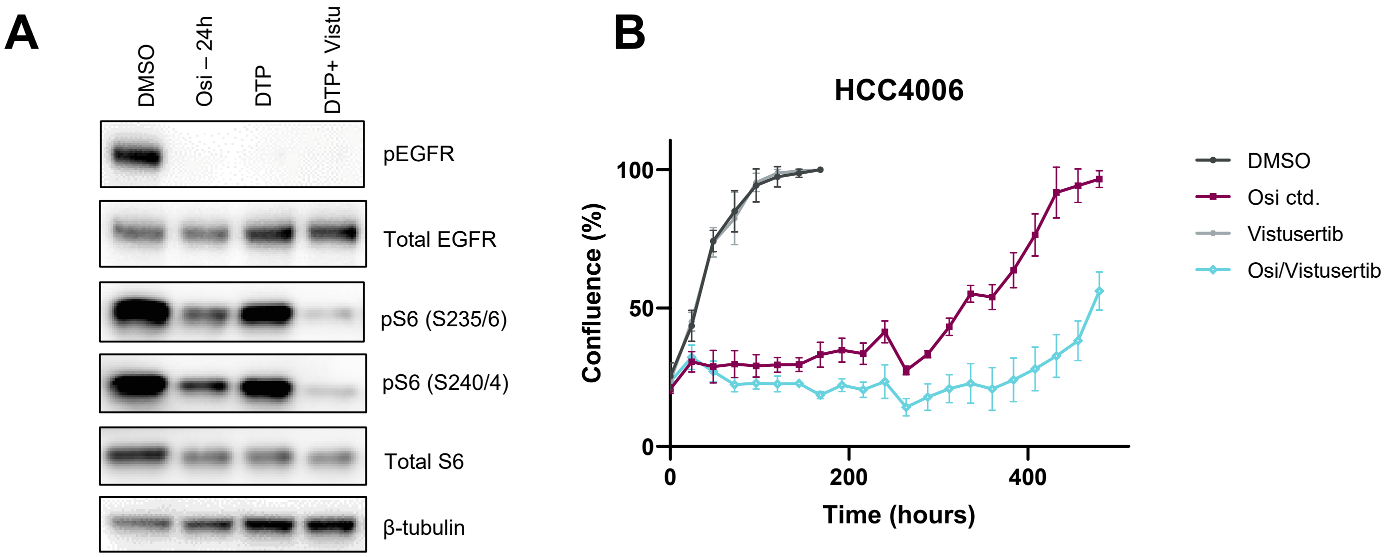


**Appendix Figure S8. Effect of mTORc1/2 inhibition on DTP growth.** (A) HCC4006 were treated with Osimertinib for 24h, 14 days (DTP) or 11 days followed by 3 days with the combination of Osimertinib + 100 nM Vistusertib (DTP + Vistu), followed by western blotting for the indicated proteins. (B) HCC4006 cells were treated continuously with Osimertinib (160 nM) Vistusertib (100 nM) or the two drugs in combination, and growth was assessed as % confluence using the Incucyte imaging platform (n=3).

# Appendix Figure S9

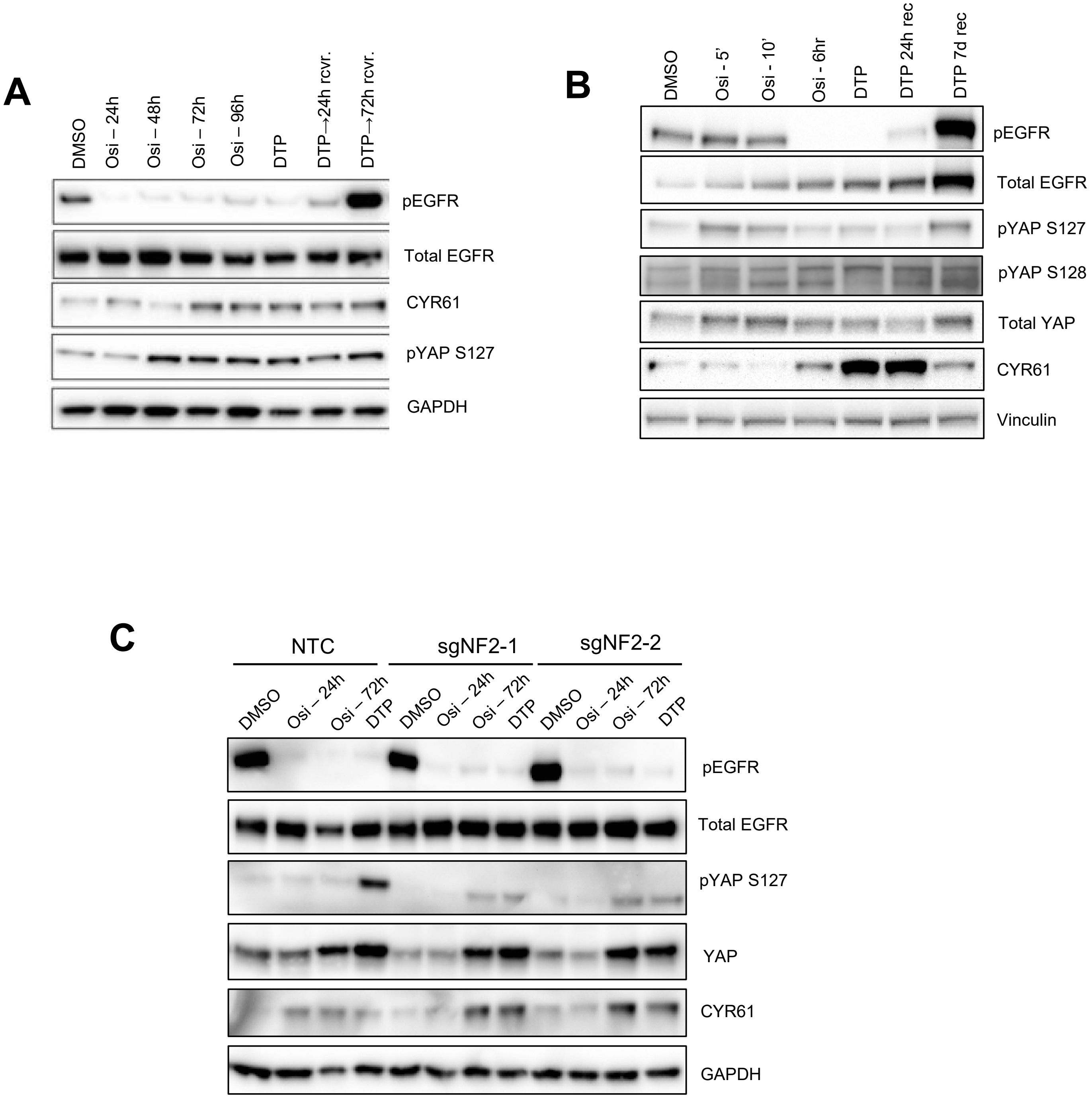


**Appendix Figure S9. Effect of Osimertinib on YAP phosphorylation and pathway output.** (A) NCI-H1975 and (B) HCC4006 cells were treated with Osimertinib for the indicated times, lysed and immunoblotted for the indicated proteins. (C) PC9 cells where NF2 had been deleted using two distinct guide RNAs (as well as non-targeting control (NTC)) were treated with Osimertinib for 24h, 72h or 14 days (DTP). Cells were then lysed and immunoblotted for the indicated proteins.

# Appendix Figure S10

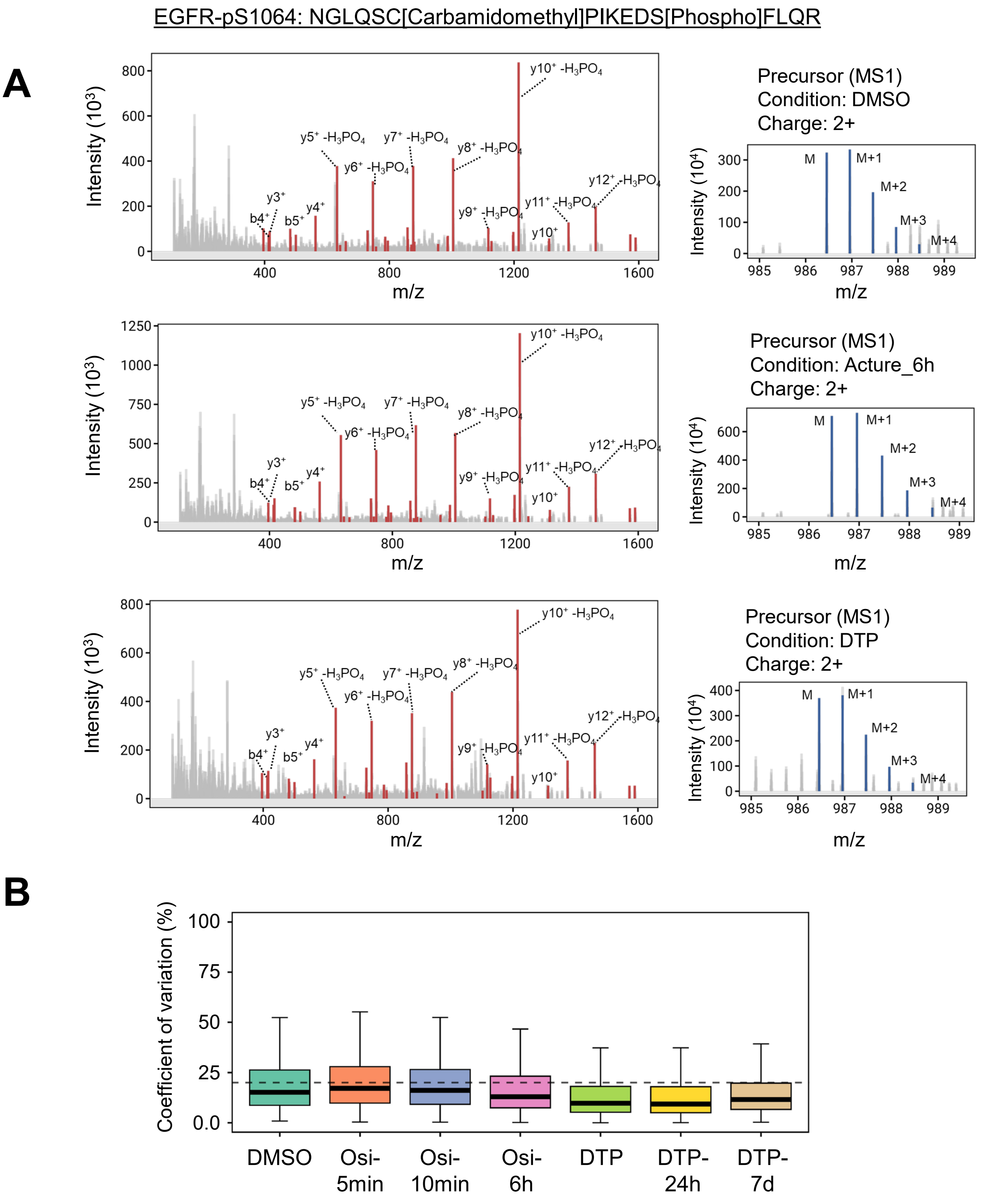


**Appendix Figure S10. Evidence for DIA Precursor Identification and Quantitative Reproducibility.** (A) Each panel displays the XICs of fragment ions corresponding to representative DIA precursors. The EGFR-S1064 phosphopeptide is shown at three time points to illustrate its quantitative profile. (B) Coefficient of variation (CV) per treatment timepoints in the DTP model. CVs below 20% were considered acceptable.
